# Opinions of Psychologists in Poland Regarding the Possibility of Prescribing Psychotropic Drugs—A Cross-Sectional Study

**DOI:** 10.3390/jcm13216560

**Published:** 2024-10-31

**Authors:** Jolanta Banasiewicz, Hanna Rozenek, Monika Kos y Gonzales, Stanisław Wójtowicz, Kornelia Zaręba

**Affiliations:** 1 Department of Health Psychology, Medical University of Warsaw, 02-091 Warsaw, Poland; jolanta.banasiewicz@wum.edu.pl (J.B.); hanna.rozenek@wum.edu.pl (H.R.); stwojt@o2.pl (S.W.); 2 Counseling and Educational Psychology, New Mexico State University, Las Cruces, NM 88003, USA; eumenides60@hotmail.com; 3 Old Polish University of Applied Sciences, 25-666 Kielce, Poland; 4 Department of Obstetrics and Gynecology, College of Medicine and Health Sciences (CMHS), United Arab Emirates University (UAEU), Al Ain, United Arab Emirates

**Keywords:** psychologists, prescribing rights for psychologists, mental health care, Poland, regulatory framework for psychologists, ethical concerns, psychopharmacology, mental health system, psychiatric medications

## Abstract

**Background/Objectives**: Discussions about the possibility of psychologists prescribing medications have been ongoing for several years. The study aims to ascertain the opinion of the Polish professional community of psychologists on the right of psychologists to prescribe psychotropic drugs. **Methods**: The study was conducted on online forums (Porozumienie Psychologów) associated with psychologists from all over the world from 15 April 2023 to 30 September 2023. The participants were asked to fill out a Google survey consisting of 26 questions. **Results**: A total of 677 psychologists participated in the study, including 580 (85.7%) women and 97 (14.3%) men. The majority of the respondents were at the peak of their life activity, between 30 and 50 years of age. A large group of respondents believed that a psychologist should have the right to prescribe psychotropic drugs (46.5%) and declared their participation in activities to promote these rights (52.9%). The vast majority of respondents reported that psychologists authorized to prescribe drugs should complete additional courses as part of the pharmacology specialization (74.8%) and should pass an exam in this field (73.4%) or should complete additional courses in the field of pharmacology (74.8%). Such opinions were much more common in the group with psychological specializations. In this group, more people allowed for such a privilege for those who have completed studies, have documented five years of experience, or have a psychotherapist certificate (*p* < 0.0001). **Conclusions**: There may be many societal needs that could be successfully met by psychologists obtaining prescriptive privileges. However, psychologists ought to understand that our obligations need to transcend guild concerns and appropriate qualifications.

## 1. Introduction

Discussions about the possibility of psychologists prescribing medications have been ongoing for several years [1]. This topic has been discussed many times on the forum of the Polish Trade Union of Psychologists and has both staunch supporters and opponents [1]. Some psychologists in online forums—especially those who do not work clinically, who are employed in education, social care, or those dealing with occupational psychology—are not interested in obtaining this type of qualification [2]. They consider such qualifications to be completely unnecessary and even dangerous, exceeding their professional competencies, and a potential source of conflict within the psychiatrists’ community [3]. In turn, a fairly large group of psychologists with experience in healthcare and/or who hold the title of specialist in clinical psychology are in favor of allowing for such a possibility, provided that certain conditions regarding the psychologist’s appropriate education are met and that a selected group of medications are used [2].

Several countries have explored the possibility of psychologists prescribing medications [4,5,6]. This issue is specific to other countries, such as the United States of America, because questions concerning the prescriptive rights of psychologists have been explored there for 25 years [4,5,6]. Prescriptive rights for psychologists in Poland have not yet been studied in Poland through research studies, as this is a novel concept. Canadian psychologists (n = 591) and psychology students (n = 272), who were asked for their opinions on the right of psychologists to prescribe medications, in most cases (179 students and 429 professionals) indicated that appropriately trained psychologists should be able to prescribe drugs and that the Canadian Psychological Association (CPA) should advocate for such privileges [4]. Few people believed that prescribing drugs was theoretically or philosophically inconsistent with the field of psychology or that it jeopardized service-delivery psychology. Currently, psychologists are still not allowed to prescribe medications in Canada [4]. Previously, in the 1990s, there was a debate in South Africa about granting psychologists the right to prescribe psychotropic drugs (RTP) [5]. The main reasons for psychologists being awarded RTP is that it has the benefit of providing integrated treatment, with psychologists being well-placed to offer such treatment, and that there is a shortage of mental health professionals in South Africa. If psychologists received RTP, they would be required to undergo extensive training in psychopharmacology [5]. Opposition from outside the psychology field was largely based on considerations of safety related to the lack of appropriate training among psychologists [5]. According to Mureriwa et al. (2022), currently, psychologists are not allowed to issue prescriptions in South Africa; however, voices are still involved in lobbying for this right for psychologists [6]. The above article cites arguments in favor of the right to prescribe medications by psychologists. The arguments cited include the argument that ‘Training psychologists is desirable and can be done safely and effectively’. Prescribing psychologists would have appropriate training in both psychotherapy and psychopharmacology to provide integrated and comprehensive mental health interventions at lower costs.

The role of a psychologist in prescribing medications has also been discussed in Australia for some time. According to Ganguly et al., the extension of prescribing rights is already in place for optometrists, podiatrists, and nurses, which results in faster and better access to medicines for patient access to healthcare [7]. Some believe that psychologists may be involved in some form of prescribing alongside providing psychological therapy [7]. Moreover, in a survey conducted among members of the New Zealand Society of Psychology and members of the New Zealand College of Clinical Psychologists, more than half of the 571 respondents (53%) indicated that they supported the idea of prescribing medications by psychologists, and 34% of these respondents supported it under certain conditions. Certainly, the possibility of psychologists prescribing medications could shorten waiting times for patients to see psychiatrists, whose access is sometimes limited. Moreover, a visit to a psychiatrist is perceived negatively and is sometimes perceived as stigmatizing by patients and the people around them. In turn, barriers for psychologists may include insufficient knowledge in the field of pharmacology, an inability to monitor drug levels, the inability to order additional tests, such as ECGs, and a lack of ability to interpret test results. In addition, psychologists’ excessive focus on medical issues could perhaps impoverish their understanding of psychological issues; the use of effective medications would reduce the patient’s motivation to take responsibility for themselves and their desire for psychotherapy. Moreover, cardinal differences in the ability of psychiatrists and psychologists to prescribe drugs are a result of the different levels of education in both groups. As part of their curriculum, medical doctors devote significantly more hours to learning subjects, such as the physiology and pathophysiology of diseases and pharmacology, with a particular emphasis on contraindications and the side effects of drugs. Certainly, the possibility of prescribing new drugs for the most severe patients with numerous diseases, in the event of adverse reactions, or the lack of a response to drugs, should remain with doctors. Therefore, the appropriate education of psychologists was considered the most important aspect of the privilege of prescribing medicines [8]. The current system of educating psychologists in Poland, unlike the American system, does not cover pharmacology issues during studies, courses, or later specializations. American prescribing psychologists, in order to be able to obtain a prescriber’s license, must earn an additional post-doctoral master’s degree emphasizing psychopharmacology and the biological bases of behavior, pass a rigorous national exam, and engage in supervised practice. The post-doctoral education and training for prescribing psychologists in the United States of America are comparable to other mid-level prescribers, such as nurse practitioners, and the knowledge and competency are comparable to psychiatrists, psychiatric nurse practitioners, and physician assistants. After additional training and supervision, psychologists are credentialed as independent prescribing psychologists, and are only allowed to prescribe psychotropic medications that are approved for the treatment of mental and emotional disorders. To maintain the prescribing psychology certificate, prescribing psychologists are required to complete additional continuing education hours in psychopharmacology.

Currently, in the Polish healthcare system, a person requiring a consultation can consult a psychologist or a psychiatrist without a referral from a family doctor. The reasons for Polish psychologists obtaining such opportunities are primarily the relatively easy access to a psychologist, while the possibility of obtaining a psychiatric consultation is much more difficult to obtain. It should also be noted that in private medical facilities, a visit to a psychologist involves much lower costs than a visit to a psychiatrist—a visit to a psychologist is, on average, half the price of a visit to a psychiatrist [9,10]. Moreover, a visit to a psychologist takes longer than a visit to a psychiatrist, especially if the consultations take place in a public facility and their cost is reimbursed by the National Health Protection Fund. It is also important to note that a psychiatric consultation may be perceived as more stigmatizing than a psychological consultation [11,12]. Due to the above, the topic of the possibility of prescribing drugs by psychologists keeps coming back in public debates—especially recently, when a new law regarding psychologists and psychotherapists was being created. Taking into account the well-being of patients, the development of the psychological community, and the experience and results of research conducted in other countries, we decided to ask Polish psychologists about their opinions in this regard.

## 2. Materials and Methods

The study aims to ascertain the opinions of the Polish professional community of psychologists on the right of psychologists to prescribe psychotropic drugs. In order to achieve the above-mentioned aim of the study, the following research questions were formulated:

1. Does the surveyed group of Polish psychologists support extending competencies to include the possibility of prescribing medications?
2. What are the opinions of respondents on the qualifications of psychologists to prescribe psychotropic drugs?
3. What are the opinions regarding the scope of powers and limitations regarding the prescription of medications by psychologists?
4. What sociodemographic variables are related to the opinions of the surveyed psychologists regarding the possibility of prescribing psychotropic drugs by psychologists (e.g., psychologist’s scientific or professional title)?
5. What is the level of respondents’ support for selected requirements applicable to psychologists authorized to prescribe medications?

### 2.1. Materials

The study was conducted in accordance with the Declaration of Helsinki, and approved by the Bioethics Committee of the Medical University of Warsaw, which took note of the information about the study and ruled that it was consistent with the principles of scientific research ethics (certificate no. AKBE/94/2023).

A preliminary study was conducted on a focus group (12 people). After reading the opinions of the above-mentioned group and introducing appropriate comments, the research tool was made available to the respondents using an online Google survey (https://docs.google.com/forms/d/1L0n6HaboriWj0HwJUJR2_91gHdyLdefg8dtnd-kKbaw/viewform?edit_requested=true (accessed on 8 August 2024)). The study was conducted using online forums (Psychologists’ Agreement created by the National Trade Union of Psychologists) associated with psychologists from all over the world. The online forum was created exclusively for psychologists; admission to the group was verified by administrators. The survey questionnaire was completed anonymously after obtaining informed consent from the participants using Google tools. The researchers did not obtain any data enabling the identification of the subjects. The study implementation period was from 15 April 2023 to 30 September 2023.

### 2.2. Study Group

The respondents in this study were psychologists who met the following criteria for inclusion in the study group:

1. Completion of master’s studies in psychology;
2. Possessing a psychology degree;
3. Agreement to participate in the project;
4. Membership of the online forum “Psychologists’ Agreement”.

The exclusion criteria were as follows:

1. Lack of membership for the online forum “Psychologists’ Agreement”;
2. Lack of a master’s degree in psychology;
3. Lack of psychological education;
4. Lack of consent to participate in the test.

### 2.3. Research Tool

The method used in the study was a diagnostic survey using the technique of an anonymous online survey (Google) as a research tool. The research questionnaire consisted of 26 questions, both closed and open; some of these concerned the socio-demographic and professional characteristics of the study group. In addition, questions regarding the potential benefits and limitations of psychologists obtaining the right to prescribe medications were included. In this respect, the opinions and research results of other researchers who undertook similar topics and research areas were taken into account [13,14,15].

### 2.4. Statistics

The analyses were performed using the IBM SPSS v. 24, 2016 statistical package. Before performing the appropriate analyses, the normality of the variable distributions was checked using the Kolmogorov–Smirnov test.

Descriptive statistics were used in the data analysis: numerical and percentage frequencies, means, and standard deviations. The chi-squared test was used to compare the distribution of results. The significance of differences between the means in the study groups was analyzed using the Student’s t test. To assess the correlation between variables, Kendall’s tau–b correlation was used. The lowest level of significance in the analyses performed was *p* < 0.05.

## 3. Results

### 3.1. Socio-Demographic Characteristics of the Study Group

A total of 677 psychologists participated in the study, comprising 580 (85.7%) women and 97 (14.3%) men. Respondents from within the psychology profession were mostly female.

The majority of the respondents were at the peak of their life activity, between 30 and 50 years of age, and lived in large cities, although other places of residence also had a large representation. The average age of the people taking part in the study was 38.25 years (SD = 7.25). The youngest respondents were 25 years old; these were people who had recently completed master’s studies in psychology. The oldest person surveyed was 79 years old, although she was of retirement age and declared professional activity. The vast majority of people surveyed were at the peak of their life activity, between 30 and 50 years of age (Table 1).

Almost all respondents (97.9%) were active in the profession of psychology during the last year and worked mainly in large cities (65.1%). More than half of the surveyed psychologists had been working in their profession for 6 to 15 years and in medical facilities for 1 to 10 years. The average work experience as a psychologist was over 11.5 years (SD = 7.15), and the average work experience in a medical facility was just over 7 years (SD = 6.97). The majority of respondents declared that their professional experience was in the field of mental health (91.7%), and only slightly over 1% of respondents had no professional experience in this area. A large population of the respondents had a master’s degree in psychology (86.6%), and 9.6% of the respondents also had a doctorate. Almost half of the surveyed psychologists had the title of psychotherapist—most often in the cognitive behavioral and psychodynamic field. More than 20% of respondents completed a specialization in clinical psychology (Table 1).

### 3.2. Opinions of Respondents on the Qualifications of Psychologists to Prescribe Psychotropic Drugs

A large group of respondents believed that a psychologist should have the right to prescribe psychotropic drugs (46.5%) and declared their participation in activities to promote these rights (52.9%). On the contrary, 39.9% of the group were against such rights, and 32.4% of those would not support activities for them (Table 2).

### 3.3. Limitations Regarding the Prescription of Medications by Psychologists

A large group of respondents (44.3%) supported the psychologist’s right to both prescribe medications and extend prescriptions for medications previously prescribed by a psychiatrist. The majority of respondents believed that restrictions on prescribing medications by psychologists should apply to patients undergoing active treatment for another serious illness (Table 3).

### 3.4. Required Qualifications for Prescription of Medications by Psychologists

The vast majority of respondents reported that psychologists authorized to prescribe drugs should complete additional courses as part of the pharmacology specialization (74.8%) and should pass an exam in this field (73.4%) or should complete additional courses in the field of pharmacology (74.8%) (Table 4).

Regardless of whether they have a scientific or professional title or not, there is a large group of psychologists who do not agree that medications should be prescribed by a psychologist. However, more people agreed that psychologists should be able to prescribe medications. Interestingly, this group included people who believe that all those who have a psychology degree (have completed studies in this field) should have such an opportunity. Psychologists with degrees more often chose to prescribe medications provided they had a PhD and specialized in clinical psychology or gave other answers not included in the survey. In both groups, a relatively large group of people made the ability to prescribe medications conditional on clinical specialization and appropriate professional experience. The differences described were significant at the *p* < 0.0001 level.

A large group of respondents (44.3%) supported the psychologist’s right to both prescribe medications and extend prescriptions for medications previously prescribed by a psychiatrist. The majority of respondents believed that restrictions on prescribing medications by psychologists should apply to people undergoing active treatment of another serious illness (Table 5).

To sum up the above results, the largest group of less-qualified (without specializations) respondents believe that psychologists should not prescribe medications. In turn, among respondents with specializations, almost 30% believe that it could be possible, but only after obtaining a clinical specialization or a psychotherapist certificate and at least 5 years of experience. The differences described were significant at the *p* < 0.0001 level.

## 4. Discussion

Although the opinion of psychologists about prescriptive rights for psychologists is a very vast and deep topic deserving of complex analyses, this body of work focuses on narrower aspects of this discussion—trends among Polish psychologists. The general trend that emerged through this research study revealed that a large number of Polish psychologists are in favor of psychologists possessing prescriptive authority and they are supporting the movement. Regardless of whether they have a scientific or professional title or not, the largest group who do not agree with the prescription of medications by a psychologist are people without advanced degrees. Interestingly, only in this group do people believe that all those with a psychology degree (who have completed studies in this field) should have such an opportunity. Psychologists with degrees were more likely to choose the option of prescribing medications provided they had a PhD and specialized in clinical psychology. It appears that psychologists with more advanced levels of training feel more comfortable with the idea of prescriptive rights for psychologists. The vast majority of respondents believed that psychologists qualified to prescribe medications should complete additional courses within the specialization field of pharmacology.

The results of multiple analyses showed that opinions about prescriptive authority for psychologists varied by professional level, licensure type, as well as years in the profession [15]. Fagan, and the data, suggest that sizable minorities, ranging from 19% (psychologists in independent practice) to 46% (for interns), expressed the wish to seek clinical proficiency if it were available to them [15]. Greater interest was also documented for recipients of PhDs/PsyDs, and among early-career psychologists compared to the recipients of PhD/PsyDs, and mid- and late-career psychologists. The interns had more interest in pursuing prescriptive authority than the training directors. Additionally, individuals with PhDs/PsyDs were more interested in pursuing prescription privileges than individuals without PhDs/PsyDs. The decreased interest among academic training directors as compared to practicing psychologists and student psychologists ought to be an object of further investigation and discussion.

The first inquiry regarding the prescription of drugs by psychologists appeared in Poland relatively recently, about 6 years ago [16]. This issue raises many contradicting emotions. It can be stated that there is no clear position in the Polish psychologists’ community on this matter. It is worth noting, however, that almost half of the psychologists who decided to answer the survey questions agreed with allowing psychologists the right to prescribe medicines. Psychologists with experience in healthcare see this need more often. More than half of the respondents felt that, if psychologists were granted such a right, they would engage in this type of activity. Issues similar to those covered in this work were examined by a team of Croatian researchers led by Ivan Zečević. The survey was conducted among 189 psychologists via closed Facebook groups and with the help of the Croatian Psychological Society. The majority of respondents (48.9%) of the Croatian survey were in favor of psychologists being given the right to prescribe psychotropic drugs. Statistically significant differences occurred between drug-prescribing advocates in terms of the right type of education, necessary knowledge, and arguments for prescribing medicines [17]. The vast majority of surveyed Polish psychologists probably were aware of problems resulting from the lack of the provision of the Act on the Profession of Psychologists. Possibly, they would like the right to prescribe medications to be given to all people who have obtained the qualifications to prescribe drugs through specialized education. Most respondents believe that training would be a good preparation for this, when carried out as part of a specialization in clinical psychology. The need for the appropriate training of psychologists before obtaining the right to prescribe was emphasized by researchers from South Africa in the late 1990s [5]. Canadian researchers also have a similar opinion, where only a few people believed that prescribing was theoretically or philosophically inconsistent with the field of psychology or that it threatened the provision of psychological services [4].

Discussions regarding the appropriate substantive preparation of psychologists and criteria that should be met by a psychologist granted the right to prescribe drugs have appeared in every country where this issue has been discussed [18]. In the USA, in states where regulations have been adopted, psychologists who want to obtain the above-mentioned right to prescribe in law must have a doctoral degree (Ph.D./Psy.D.) and the right to practice the profession. They are also required to undergo appropriate, highly detailed postdoctoral training and education [18]. In addition, a psychologist prescribing psychotropic medications is obliged to cooperate with a doctor in the treatment process [18]. The conclusions from the previously mentioned Croatian studies are similar and indicate the need to introduce a new, very detailed method of educating psychologists, and to work under the supervision of a psychiatrist [17]. Similarly, in Great Britain, in 2018, the Professional Practice Board of The British Psychological Society outlined the following three important issues: optionality (the right to prescribe medications would not apply to every psychologist, only to people who are interested in having such a right); having completed appropriate training exams; and working in interdisciplinary teams with doctors in large hospitals and medical centers [19]. Regarding the scope of powers granted to psychologists to prescribe medications such as psychotropic substances, in a study conducted by the authors of this publication, the majority of respondents believe that restrictions on prescribing psychotropic drugs should apply to the following groups of people: patients undergoing treatment and other serious diseases; pregnant women; and children. The results of the analysis of the relationship between respondents’ opinions on the right to psychologists’ prescribing varies between professionals. It can be concluded that respondents who were outside the baseline psychological education, like specialists in clinical psychology, those who have been awarded the title of PhD or professor, or those who have graduated from psychotherapy school, believe that their additional qualifications entitle them to prescribe psychotropic drugs. The overwhelming majority of surveyed Polish psychologists, most probably aware of the problems resulting from the lack of functioning of the Act on the Psychology Profession, would like to give the right to prescribe medications to a particular group of psychologists and restrict it to people who have obtained detailed, specialized education. Most respondents believe that training conducted as part of a specialization in clinical psychology would be a good preparation for this. As for the scope of powers granted to psychologists who can prescribe psychotropic drugs, the majority of respondents think that restrictions on the prescription of psychotropic drugs should apply to the following groups of people: patients undergoing treatment for other serious diseases; pregnant women; and children.

In the UK, research carried out in 2018–2020 among 20,270 adults living in Great Britain in order to assess the public perception of psychologists lobbying for the right to prescribe medicines showed that 46% of respondents support, and 15% oppose, the idea that psychologists should have the right to prescribe medications [20]. Among people who have never experienced any problems with mental health issues and have used the help of a psychologist or psychiatrist, the idea of prescribing medications by psychologists was supported by 56–57% of respondents. The dominant view among opponents was that only doctors should prescribe drugs because psychologists do not have the appropriate education in this area [20]. Apart from that, the psychologist’s task is to talk to the patient and not to prescribe medications. Supporters of granting psychologists the above right believe that such legal regulation would significantly facilitate the functioning of sick people because it would eliminate the need to visit two specialists for one disease, shorten the waiting time for medications to be prescribed, increase confidence in psychologists, and would reduce the number of medications prescribed. We are also planning a study examining the opinion of a representative sample of the Polish population on prescribing medications by psychologists.

According to Fagan et al., “The majority of respondents… believed that the prescription privileges for psychologists may facilitate a better understanding of mind-body interaction, will enhance psychologists’ clinical skills, will help improve collaboration among psychologists and other health care providers and will ultimately benefit certain subjects of the population” [15]. This study already supplies us with an understanding of what psychologists who are trained at the higher tier of the profession want—development. Fox et al. reported that “there are several reasons as to why we may have a slow rate of growth. Our profession has clear divisions between its practice and the academic branches, leading to the absence of advocacy issues- even among practitioners” [21].

In the discussion on the possibility of psychologists prescribing drugs in Poland, the fact of their complicated situation is also important. In fact, in Poland, there are no regulations authorizing psychologists to prescribe medications. In the case of representatives of other medical professions, these issues are regulated on the basis of specific acts relating to the practice of the profession, e.g., Article 45 of the Act on the Profession of Doctors and Dentists (Journal of Laws, 2024, item 1287). Article 15a of the Act on the Nursing and Midwifery Professions (Journal of Laws, 2022, item 2702) indicates the right of nurses and midwives to prescribe certain groups of drugs and issue prescriptions for them. In this context, an important argument raised against psychologists obtaining the right to prescribe psychotropic drugs is the lack of an actively operating act for the psychology profession. The current act was passed on 8 June 2001 and entered into force in 2006 [22]. However, this Act was never applied due to legislative errors. No implementing provisions were established in its scope. There has never been a professional self-government to decide whether graduates of psychology should be admitted to practice their profession. The Act is also inconsistent with European Union law in the area of mutual recognition of professional qualifications of psychologists by EU countries [23,24]. Currently, mainly thanks to the activities of the Polish Trade Union of Psychologists, the Ministry of Health, and the Polish Psychological Association, work on a new act for the profession of psychology is being finalized. The new act will allow for the establishment of professional self-government for psychologists and will enable the regulation of the issue of obtaining the right to practice the profession of psychology [25]. Due to the lack of an actively operating act, in practice, psychological services in Poland can be provided by any person (even those who have no psychological training) [23]. The lack of an actively functioning act in the profession of psychology does not allow for reliable information on the number of psychologists who have completed psychology studies and have started and continued to work in their profession to be obtained. There is also no reliable information regarding the number of psychologists working in healthcare. At the same time, an increasing number of psychologists are acquiring broader professional qualifications by obtaining the state title of specialist in clinical psychology or obtaining a psychotherapist certificate. Because of the lack of an active law, psychologists are currently prevented from obtaining the right to prescribe psychotropic drugs.

There are arguments for and against prescription privileges for psychologists. This debate reflects a deep schism in psychology that represents a disunity in the field. This disunity is seen as a divide between those trained to be psychotherapists and those trained to be scientist-practitioners. It is argued that those in the former group support prescriptive privileges and are interested in the survival of professional schools, while the latter oppose prescriptive privileges and are interested in the survival of university departments of psychology [2]. For the discipline to survive, there must be a rapprochement between these factions and alternatives. We believe that before implementing such a law, it is necessary to reform the education of psychologists before they will be allowed to prescribe medication. A large part of the Polish psychology community is afraid of negative reception and criticism from the medical community. The point is not for psychologists to replace doctors, but for them to constantly develop within their profession, just as the nursing professional has evolved from a person performing care functions into a thoroughly educated specialist who also has diagnostic and therapeutic capabilities. The education of a psychologist, even after additional training, will not be comparable to that of a doctor. There are also voices pointing out the danger of the medicalization of the psychologist’s profession, which may result in pharmaceuticals being chosen instead of psychotherapy as a faster way to achieve improvement, especially with the constantly developing marketing of pharmaceutical companies [2].

Psychology is a relatively young profession and practitioners need to evaluate the maximum abilities of the scope of their practice. Several psychologists have already embraced psychopharmacology without safety difficulties. There may be many societal needs that could be successfully alleviated by psychologists obtaining prescriptive privileges. Over time, well-founded ideas evolve or become obsolete. We need to examine our priorities and strategies in the context of societal needs, and the political landscape, both internal and external, if we are to ensure the success of prescriptive authority. We are just a small band on the wide spectrum of physical and mental professions, not intending to encroach on anyone’s field by filling the gap of vastly needed help. By enhancing our capability and broadening our training and understanding of mind–body interactions, we have a chance to grow and develop as professionals.

### 4.1. Limitations

Our research has some limitations that can influence the results, including some online questionnaire biases such as sampling bias. The study only covers a group of psychologists who are members of an online group, which is not a representative group for the entire country. Moreover, some nonresponse biases may have occurred, e.g., the survey did not include older psychologists who do not use social media. Similarly, the studied group, although large, constitutes only a small percentage of all psychologists in Poland, whose current number we estimate at approximately 15,000. Unfortunately, due to the lack of regulations in this area, we do not have specific numerical data and access to all psychologists. This problem might also be a major limitation in introducing medication prescriptions by psychologists. Moreover, social desirability bias may have occurred, most probably in the form of over-reporting, because of psychological societal expectations.

### 4.2. Strengths

To our knowledge, this is the first study of this type conducted in Poland. The presented study involved psychologists recruited from various areas of professional experience and different parts of the country, which guarantees the diversity of the study group. The formula of the survey, which also included open questions, additionally enabled participants to freely express their opinions.

## 5. Conclusions

The continuous evolution and expansion of psychological practice requires periodic changes, self-reflection, and reassessment of goals. There is an agreement that not all psychologists agree with the idea of receiving psychopharmacology training, and this point of view should be respected and honored. However, there is a consensus amongst those who seek re-specialization that psychopharmacology training needs to occur at the postdoctoral level. Prescribing psychologists offer a unique set of skills and enhancements that psychiatrists may be lacking—such as conciliary or broader training in humanistic approaches towards human beings compared to the linear scientific and medical training of psychiatrists. We offer a unique perspective and ought not to be regarded in terms of dualistic black-and-white judgments coming from both sides and from within. If a system were established that enabled psychological professionals to prescribe medications, it would complete the grey gap between psychotherapy and drug therapy.

There is an increasing global mental health crisis and a shortage of psychiatrists to meet the demand. Rural, urban, and suburban areas all struggle to provide sufficient psychiatric services to patients. Most psychotropic medications are currently prescribed by primary healthcare professionals, including physicians and nurses; however, these professionals often have limited training in mental health treatment. Prescribing psychologists can increase patient access to psychotropic medications, decrease wait times, and safeguard better follow-up care for patients already on psychotropic medications. Moreover, easier access to the possibility of continuing psychiatric treatment will reduce the frequency of complications related to drug withdrawal due to financial constraints and the possibility of obtaining a quick psychiatric consultation. American prescribing psychologists have proved that prescribing psychologists can manage medication treatment for most mental health disorders successfully and safely; therefore, other countries, such as Poland, can use that example to lead their efforts toward prescriptive rights for psychologists.

## Figures and Tables

**Table 1:** Characteristics of the study group, including sociodemographic variables and professional functioning.

Demographic Characteristic		N	%
Professional life activity	25–30	87	12.9
	31–40	370	54.7
	41–50	182	26.9
	51–60	31	4.6
	Over 60	7	1.0
Place of residence	Village	69	10.3
	City up to 50,000 inhabitants	94	14.0
	City between 50–150 inhabitants	79	11.7
	City between 150–500,000 inhabitants	140	20.8
	City above 500,000 inhabitants	291	43.2
Place of primary employment in the profession of psychology	Village	27	4.0
	City has 50,000 inhabitants	102	15.1
	City between 50–150,000 inhabitants	98	14.5
	City between 150–500,000 inhabitants	143	21.1
	City above 500 000 inhabitants	298	44.0
	Lack of data	9	1.3
Experience in the profession of psychology	0	5	0.7
	1–5	138	20.4
	6–10	198	29.2
	11–15	173	25.5
	16–20	100	14.8
	21–25	37	5.5
	26–30	11	1.6
	Above 30	13	1.9
	Lack of data	2	0.3
Work experience in medical facilities	0	111	16.4
	1–5	240	35.5
	6–10	139	20.5
	11–15	99	14.6
	16–20	48	7.1
	21–25	17	2.5
	26–30	7	1.0
	Above 30	4	0.6
	Lack of data	12	1.8
How extensive is your professional experience in the area of mental health??	1 (Not at all)	9	1.3
	2	13	2.0
	3	34	5.0
	4	99	14.6
	5 (All of it)	522	77.1
Scientific title	Doctor of Humanities	20	2.9
	Doctor of Medical Sciences	24	3.5
	Doctor of Health Sciences	19	2.8
	Doctor of Social Sciences	23	3.3
	Doctor of Philosophy	8	1.1
	Associate Professor	1	0.1
	Full Professor	1	0.1
	Master of Psychology	584	86.8
Professional interest	Psychologist	674	99.6
	Clinical psychologist	145	21.4
	Psychotherapist	337	49.8
	Addictions	7	1.0
	Cognitive	141	20.8
	Schema	1	0.1
	Gestalt	3	0.4
	Psychodynamic	145	21.4
	Systems	42	6.2
	Humanistic	10	1.5
	Integrative	31	4.6
	TSR	4	0.6
	Other	2	0.2

**Table 2:** Opinions of respondents on the qualifications of psychologists to prescribe psychotropic drugs.

		Definitely No	No	Do Not Know	Yes	Definitely Yes
Do you think that a psychologist should have the license to prescribe psychotropic drugs?	N	174	96	92	133	182
	%	25.7	14.2	13.6	19.6	26.9
Would you be engaged in the possibility of obtaining authorization to prescribe medicines by psychologists if this possibility appeared?	N	149	69	102	102	255
	%	22.0	10.2	15.1	15.1	37.7

**Table 3:** Opinions regarding the scope of powers and limitations regarding the prescription of medications by psychologists—frequency of respondents.

		N	%
Do you think that:	A psychologist should have the right to prescribe medications	40	5.9
	A psychologist should only have the right to extend prescriptions for drugs previously prescribed by a psychiatrist	143	21.1
	A psychologist should have the right to both prescribe medications and extend prescriptions for medications previously prescribed by a psychiatrist	300	44.3
	A psychologist should not be allowed to prescribe any medications	169	25.0
	Other	25	3.7
If psychologists are allowed to prescribe medicines, which patient groups should be excluded from this privilege?	Children	394	58.2
	Adolescents	250	36.9
	People over 65 years of age	223	32.9
	Pregnant women	434	64.1
	People with diagnosed mental disorders	156	23.0
	People with serious somatic diseases	430	63.5
	People with intellectual disabilities	154	22.7
	People were undergoing active treatment for another serious illness	478	70.6
	Other	31	4.6

**Table 4:** Level of respondents’ support for selected requirements applicable to psychologists authorized to prescribe medications.

		Definitely Yes	Yes	Do Not Know	No	Definitely No	Lack of Data
Psychologists licensed to prescribe medications should complete additional courses within the pharmacology specialization	N	431	75	62	26	81	2
	%	63.7	11.1	9.2	3.8	12.0	0.3
Psychologists licensed to prescribe medications must first pass a pharmacology exam	N	430	68	62	32	83	2
	%	63.5	10.0	9.2	4.7	12.3	0.3

**Table 5:** Opinions regarding the authorization to prescribe psychotropic drugs depending on the psychologist’s scientific or professional title.

The Right to Prescribe Medicines Should Have an Academic Title of:			Scientific Title		Specialization	
			No	Yes	No	Yes
All people with the title of psychologist		N	5	0	3	2
		%	0.9	0.0	0.6	1.4
Psychologists who have completed five-year uniform master’s studies in psychology		N	34	0	34	0
		%	5.8	0.0	6.4	0.0
Psychologists who specialize in clinical psychology		N	96	12	66	42
		%	16.4	13.3	12.4	29.2
Psychologists who hold a PhD		N	12	1	12	1
		%	2.0	1.1	2.3	0.7
Psychologists who hold a PhD and specialization in clinical psychology		N	16	11	17	10
		%	2.7	12.2	3.2	6.9
Psychologists with a documented minimum of five years of experience in providing mental health services		N	59	8	61	6
		%	10.1	8.9	11.5	4.2
Psychologists with a documented minimum of five years of experience in providing mental health services and specialization in clinical psychology		N	92	11	69	34
		%	15.7	12.2	13.0	23.6
Psychologists certified as a psychotherapist		N	39	4	41	2
		%	6.7	4.4	7.7	1.4
No psychologist should be allowed to prescribe drugs		N	166	22	154	34
		%	28.3	24.4	28.9	23.6
Other		N	67	21	75	13
		%	11.4	23.3	14.1	9.0
	Chi Square		34.636		60.620	
	*p*		0.0001		0.0001	

## Data Availability

The original contributions presented in the study are included in the article, further inquiries can be directed to the corresponding authors.
